# Advanced therapies and cancer policy in Latin America: why regulatory coherence matters more than technological capacity

**DOI:** 10.3389/fpubh.2026.1777671

**Published:** 2026-02-05

**Authors:** Evelyn Silva-Moreno

**Affiliations:** Fundación Arturo López Pérez OECI Cancer Center, Centro de Investigación e Innovación en Cáncer, Santiago, Chile

**Keywords:** advanced cancer therapies, equity in cancer care, health system sustainability, mission-driven institutions, regulatory governance

## Introduction

Across Latin America, advanced therapy medicinal products (ATMPs)—particularly cell- and gene-based interventions—have increasingly been portrayed as symbols of biomedical progress, innovation, and scientific leadership in oncology (1, 2). However, their gradual incorporation into national health systems has revealed a persistent governance paradox: technological capabilities have advanced more rapidly than the ethical, regulatory, and institutional frameworks required to steward these therapies responsibly within public health systems.

This Opinion article argues that the principal determinant of whether ATMPs contribute to equitable, patient-centered cancer care is not technological sophistication or industrial scale, but rather regulatory coherence, institutional alignment, and a clearly defined social mandate guiding innovation. While advanced therapies are often discussed through the lenses of scientific advancement or clinical novelty, their real-world impact in oncology is fundamentally shaped by governance structures, regulatory capacity, and health system priorities.

Cancer care—particularly in the context of high-cost, high-complexity interventions such as ATMPs—demands regulatory systems capable of ensuring patient safety, ethical oversight, and equitable access. In Latin America, marked differences in regulatory maturity, enforcement capacity, and alignment with national cancer policies critically determine how innovation is translated into population-level health benefit. Consequently, any meaningful discussion of advanced cancer therapies must explicitly address the policy and regulatory architectures that enable—or constrain—their responsible integration into public health systems.

## Beyond technological optimism: structural tensions in Latin America

The regional policy environment for ATMPs in Latin America remains highly heterogeneous. Countries with consolidated regulatory authorities and coordinated public research programs have made progress toward supervised clinical translation, whereas others continue to face fragmented enforcement, regulatory ambiguity, and weak institutional coordination. Three cross-cutting tensions shape this landscape: (i) technological sovereignty vs. health system sustainability; (ii) innovation vs. equity in access and financing; and (iii) scientific credibility vs. commercial opportunism, particularly in settings where unproven cellular interventions proliferate within regulatory gaps.

These tensions illustrate a critical public health lesson: innovation without governance does not constitute progress. Current regulatory frameworks for cancer-related advanced therapies vary substantially across the region. Brazil operates under a relatively consolidated model led by ANVISA, which has established specific regulatory pathways for ATMPs, including requirements for clinical-grade manufacturing, traceability, and post-authorization surveillance (3, 4). Argentina has developed supervised translational pathways through CONICET-linked public–academic programs, integrating cell and gene therapies within hospital-based research environments under defined ethical and regulatory oversight (5, 6).

In contrast, Mexico exemplifies a more fragmented regulatory landscape, where federal norms coexist with limited enforcement capacity. This has created regulatory gray zones that have facilitated the commercialization of unproven cellular interventions marketed directly to cancer patients (7, 8). Chile has adopted a more precautionary and governance-centered approach, with the Instituto de Salud Pública (ISP) defining explicit regulatory criteria for advanced therapies and aligning their evaluation with the National Cancer Plan, despite the country's limited industrial manufacturing capacity (9, 10).

Taken together, these national experiences demonstrate that governance coherence—rather than technological capability alone—is decisive for patient protection, public trust, and system legitimacy (4, 11). The central challenge for ATMPs in Latin America is therefore not a lack of scientific expertise, but the absence of harmonized governance ecosystems capable of aligning innovation with cancer control priorities. Without regulatory coherence, ethical oversight, and sustainable financing mechanisms, advanced therapies risk reinforcing existing structural inequalities rather than contributing to equitable cancer care (11, 12).

## Mission-driven institutions and social purpose in cancer innovation

Mission-driven institutions play a pivotal role in mediating between innovation, regulation, and social purpose (12). The experience of the Centro de Investigación e Innovación en Cáncer (CIIC-FALP) illustrates how advanced therapies can be embedded within an institutional model that integrates clinical care, research, and public accountability. Importantly, this approach depends not only on institutional leadership, but also on the active involvement of clinicians and policymakers as co-stewards of innovation.

Clinicians function as gatekeepers of ethical implementation, ensuring that advanced therapies are introduced within evidence-based protocols and aligned with patient-centered values. Policymakers, in turn, define the regulatory, legal, and financing conditions that determine scalability, sustainability, and equity. When these actors operate in isolation, innovation risks becoming disconnected from population health needs. Conversely, when clinicians, regulators, and decision-makers collaborate within mission-driven institutions, advanced therapies can be assessed not only in terms of clinical efficacy, but also with respect to social value, health system sustainability, and contribution to national cancer control strategies.

Rather than treating ATMPs as isolated high-cost technologies, CIIC-FALP embeds them within a continuum of care that includes clinical treatment, research, psychosocial support, and long-term patient accompaniment. In this model, innovation is evaluated not solely through traditional clinical endpoints, but also through its contribution to dignity, quality of life, and the reduction of structural inequities. From this experience, four policy priorities emerge for the region: (1) regulatory coherence as a public health safeguard; (2) robust and transparent ethical oversight systems as foundations of institutional trust; (3) context-adapted evaluation models integrating clinical outcomes with sustainability and equity considerations; and (4) implementation pathways explicitly aligned with national cancer control priorities.

These governance challenges are further compounded by macroeconomic constraints. In Mexico, public expenditure on health as a proportion of gross domestic product has declined over recent years, intensifying pressures on an already fragmented cancer care system and limiting the capacity for sustained investment in regulated innovation. Under such conditions, advanced therapies risk exacerbating inequities, as access becomes increasingly mediated by private markets rather than public health priorities. This underscores the need to analyze cancer innovation not only through regulatory lenses, but also through fiscal and health financing frameworks.

## Conclusion

Latin America stands at a strategic crossroads. The region may continue as a passive recipient of externally developed advanced cancer therapies, or it may consolidate regulatory clarity, institutional alignment, and ethical stewardship as the true infrastructures of responsible innovation. We argue that meaningful progress in ATMPs begins not with technological scale, but with social purpose embedded in public health governance.

From a public health perspective, the challenge is not to accelerate the adoption of advanced cancer therapies at any cost, but to govern their integration responsibly. The Latin American experience demonstrates that regulatory coherence, institutional mission, and cross-sector dialogue among clinicians, policymakers, and researchers are more decisive than technological capacity alone. Under coherent governance frameworks, advanced therapies can strengthen cancer systems and reduce inequities; without them, they risk amplifying existing structural vulnerabilities.
